# Antioxidant properties of water-soluble polysaccharides prepared by co-culture fermentation of straw and shrimp shell

**DOI:** 10.3389/fnut.2022.1047932

**Published:** 2022-11-21

**Authors:** Yongmei Lyu, Mian Wang, Yiwen Zhang, Xiaoyang Zhang, Xiaochen Liu, Fengwei Li, Dujun Wang, Ming Wei, Xiaohong Yu

**Affiliations:** ^1^School of Marine and Bioengineering, Yancheng Institute of Technology, Yancheng, China; ^2^Jiangsu Key Laboratory of Marine Bioresources and Environment, Jiangsu Ocean University, Lianyungang, China

**Keywords:** polysaccharide, straw, shrimp shell, co-culture fermentation, antioxidation

## Abstract

Herein, we present a method for producing water-soluble polysaccharides (WSPs) by co-culture fermentation of straw and shrimp shells. The chitin-degrading strain was isolated and genotypically identified as the non-pathogen *Photobacterium* sp. LYM-1 in this study. *Photobacterium* sp. LYM-1 and *Aureobasidium pullulans* 2012 could coexist without antagonism. WSPs concentrations were higher in co-culture fermentations of *Photobacterium* sp. LYM-1 and *A. pullulans* 2012 (PsL/AP-WSPs) compared to monocultures (PsL-WSPs and AP-WSPs). FTIR was used to examine the polysaccharide properties of three WSP fractions. The monosaccharide compositions of three WSPs fractions were primarily composed of mannose, ribose, glucosamine, glucose, galactose, and arabinose with varying molecular weights and molar ratios according to HPLC analysis. PsL/AP-WSPs showed better scavenging effects on DPPH, ABTS, and OH free radicals, demonstrating the application potential of PsL/AP-WSPs from straw and shrimp shells. The maximum yield obtained under optimum conditions (fermentation time of 6 days, temperature of 31°C, inoculum concentration of 10% (w/v), and inoculum composition of 2:1) was 5.88 ± 0.40 mg/mL, based on the PsL/AP-WSPs production optimization by orthogonal design. The results suggest that an environmentally friendly approach for WSPs production from agro-food wastes straw and shrimp shells was developed.

## Introduction

Polysaccharides play multiple roles in the life process with extensive biological activities and a huge potential in the fields of pharmaceutical, food, and cosmetics industries due to their efficacy and relatively low toxicity. Natural polysaccharides exhibited diverse bioactivities, such as antioxidant (1), anti-inflammatory (2, 3), antitumor (4), antiobesity (5), immunomodulatory (6), hypoglycemic (7), and prebiotics activities (8). Natural polysaccharides are typically extracted using various techniques or fermented by microbes. Hot-water extraction (HW), acid/alkali extraction (9), ultrasonic-assisted extraction (UA), microwave-assisted extraction (ME) (10), enzyme-assisted extraction (EA), and enzyme-ultrasonic assistance extraction (EUA) (11) are the most commonly used extraction techniques today. While each technique has advantages and disadvantages, the efficiency of polysaccharides extraction, time consumption, cost, and environmental impact must all be considered. During the preparation, the physiochemical and biological function of polysaccharides changed (12). Microbial polysaccharides produced by a wide range of microorganisms are generally water-soluble polysaccharides (WSPs) and the final products are more quality controlled (13). Microbial fermentation is a relatively green and environmentally friendly method of polysaccharide preparation when compared to other methods. They are gaining more recognition in the fields of food and Pharmaceuticals. Pullulan is a microbial polysaccharide produced by the yeast-like polymorphic fungus *Aureobasidium pullulans* (14).

Microbial exopolysaccharides (EPSs) include both homopolysaccharides and heteropolysaccharides. Pullulan is a type of homopolysaccharide, whereas xanthan and gellan are typical heteropolysaccharides. Microbial EPS are widely used in the development of functional foods (15). Submerged fermentation (SmF) and solid-state fermentation (SSF) are widely used two methods for the production of EPS. Many strategies have been implemented to improve the yield and titer of microbial EPS production during batch fermentation (16–18). However, the key factor restricting the wider application of EPS is the high cost of mass production. Therefore, cheap substrates for reducing the cost to facilitate applications of EPS are a practical approach (19). Many agro-food wastes, such as potato waste, de-oiled rice bran, jackfruit seeds, the hull hydrolysate, and bran of rice and wheat, have been used as materials for the production of EPSs by *A. pullulans* (20, 21). Straw is one of the richest agro-food wastes in the world, which is a potential candidate for the production of EPS by *A. pullulans* (22).

The disposal of a vast quantity of shrimp shell waste has become a major ecological and environmental issue. Even efforts to extract chitin and chitosan from shrimp shell waste *via* chemical processes or fermentation can result in environmental pollution (23). Acid and alkali treatments have to be used during the fermentation process for water-insoluble chitin or chitosan preparation from shrimp shells (24). In addition to chitin and chitosan, studies on the production of various bioactive metabolites by the fermentation of shrimp shells with different strains have been reported (24, 25). To the best of our knowledge, the exopolysaccharides preparation by the fermentation of chitin-degradation strain *Photobacterium* sp. has not been reported.

This work aims to obtain WSPs by co-fermenting straw and shrimp shell wastes. In this study, shrimp shell degradation strain was isolated and identified first using chitin as the index. To economically utilize these agro-food wastes, the compatibility between the straw-degradation strain and the shrimp shell degradation strain was further investigated. The molecular weight, monosaccharide compositions, chemical properties, and antioxidant activity of WSPs were analyzed. Finally, the fermentation conditions to produce WSPs were optimized. Our findings could be useful in the development of novel exopolysaccharides from low-cost substrates.

## Materials and methods

### Bacterial strains, chemicals, and media

Rice straw and shrimp shells were purchased from Yancheng Farmers Market, Jiangsu Province, China. *Photobacterium* sp. LYM-1 was isolated from mudflat collected in Yancheng coastal mudflat areas, Jiangsu, China. *Aureobasidium pullulans* 2012 was obtained from the College of Food Science and Technology, Nanjing Agricultural University, China, originally isolated from the food environment. All reagents and chemicals used in this study were of the highest grade available from the suppliers, except for the HPLC solvents (LCMS grade).

Liquid enrichment medium (g/L): chitin powder 8.0, (NH_4_)_2_SO_4_ 2.0, natural pH, sterilized at 121°C for 20 min.

Colloidal chitin agar (g/L): colloidal chitin 4.0, (NH_4_)_2_SO_4_ 2.0, agar 15.0, sterilized at 121°C for 20 min.

Seed medium: Luria–Bertani (LB) medium (g/L): yeast extract 5, tryptone 10, sodium chloride 10, natural pH, sterilized at 121°C for 20 min.

Yeast extract peptone dextrose (YPD) culture medium (g/L): tryptone 20, yeast extract 10, glucose 20, natural pH, sterilized at 121°C for 20 min.

Fermentation medium (g/L): straw 15, shrimp shell 30, natural pH, sterilized at 121°C for 20 min.

### Screening, identification, and compatibility experiment of strains

#### Isolation of chitin-degrading bacteria

The samples were collected from Yancheng coastal mudflat areas of Jiangsu province, China. Nine soil samples (5 g) were diluted with 25 mL of sterile water in 50 mL centrifuge tubes and vortexed. Five milliliters of diluents were inoculated into an enrichment medium and incubated at 30°C for 48 h. Then, 500 μL of enriched culture was smeared onto the colloidal chitin agar petri dish with 10^–4^, 10^–5^, and 10^–6^ times dilution, respectively, and incubated at 28°C for 72 h. The target isolate was identified by the chitin hydrolysis zone.

#### Identification of chitin-degrading bacteria by 16S rDNA amplification

The genomic DNA of the best isolate was prepared using the Vazyme bacteria DNA isolation mini kit (DC112-01). Polymerase chain reaction (PCR) amplification of the 16S rRNA gene sequence was performed for species-level detection. The oligonucleotide primers for amplifying 16S rRNA were as follows: forward 27F: (5′ AGAGTTTGATCCTGGCTCAG 3′) and reverse primers 1492R: (5′ GGTTACCTTGTTACGACTT 3′). Purified PCR fragment was sequenced by DNA sequencing (Sangon Biotech, China) and compared with available 16S rRNA in the National Center for Biotechnology Information (NCBI) GenBank database.^1^ The information for the similarity rate and GenBank accession number was obtained. The MEGA7.0 software was used to construct the phylogenetic tree by the neighbor-joining technique.

#### Compatibility analysis of *Photobacterium* sp. LYM-1 and *Aureobasidium pullulans* 2012

The compatibility of the yeast strain and bacteria strain was tested using the previous method (26) with some modifications. In one group, yeast (*A. pullulans* 2012) was smeared on the surface of the YPD petri dish and bacteria (*Photobacterium* sp. LYM-1) were inoculated in the center. For another group, *Photobacterium* sp. LYM-1 was smeared on the surface of the LB petri dish and *A. pullulans* 2012 was inoculated in the center. The plates were incubated at 28°C for 48 h and the zone of inhibition was observed and recorded.

### Preparation, separation, and purification of water-soluble polysaccharides

#### Production of water-soluble polysaccharides by co-fermentation of straw and shrimp shell

Separate seed cultures of two strains were prepared by inoculating 10 μL of frozen glycerol stock into 20 mL of seed medium and incubated at 30°C, 200 rpm for 24 h. Batch fermentation was carried out by inoculating 5 mL of seed culture into 100 mL of fermentation medium and incubating at 28°C, 200 rpm for 72 h. Monocultures and co-culture fermentation groups of *Photobacterium* sp. LYM-1 and *A. pullulans* 2012 were set up (named PsL-WSPs, AP-WSPs, and PsL/AP-WSPs, respectively). The supernatant containing WSPs was collected by centrifugation at 10,000 g for 20 min.

#### Preliminary purification of water-soluble polysaccharides

After collection, the one-quarter volume of the primary crude WSPs solution was obtained by vacuum evaporation at 50°C. To precipitate WSPs, three times the volume of ethanol was added and kept at 4°C for 12 h. Subsequently, the precipitates were obtained by centrifugation at 10,000 g for 20 min and washing with absolute ethanol once more. The crude WSPs were obtained. The protein was then removed using the Sevage reagent (chloroform: normal butanol, 4:1, *v*/*v*) method (27). The resulting aqueous phase was dialyzed for 3 days using a dialysis tube (Solarbio, MWCO 8,000–12,000 Da), concentrated, and lyophilized, yielding the three WSPs fractions (PsL-WSPs, AP-WSPs, and PsL/AP-WSPs).

#### Determination of chemical composition of water-soluble polysaccharides

The polysaccharides content was determined by the phenol–sulfuric acid method with glucose (Glc) as the standard (28). The content of ash was determined as described in Shi et al. (29). The content of protein was determined with bovine serum albumin as the standard using the Bradford method (30). The content of total polyphenol was conducted by the previously reported method of Banik et al. with some modifications (31). The content of total flavonoids was determined following the method of Maina et al. with rutin as the standard (32), and the content of uronic acid was determined with galacturonic acid (GalA) as the standard as described by Blumenkrantz and Asboe-Hansen (33).

#### Molecular weight distribution determination of water-soluble polysaccharides

The molecular weight of WSPs was determined using an Agilent 1260 high-performance liquid chromatographic (HPLC) instrument (Agilent Technologies, Santa Clara, CA, USA) equipped with a column (7.8 mm × 300 mm, Tosoh Crop., Tokyo, Japan), at a constant flow rate of 0.7 mL/min and a refractive index detector (RID). The mobile phase was K_2_HPO_4_ (0.03 mM) and the sample injection volume was 18 μL. The temperature of the column and detector was 37°C. The molecular weights of WSPs were estimated by reference to the calibration curve made from Dextran standards of series known molecular weight (5, 12, 25, 50, 80, and 150 kDa).

#### Monosaccharide composition determination of water-soluble polysaccharides

The monosaccharide composition of WSPs was determined using HPLC following a previously reported method with minor modifications (34). A 20 mM mother monosaccharide standard was prepared which consists of galactose (Gal), mannose (Man), fucose (Fuc), glucosamine (GlcN), Glc, GalA, rhamnose (Rha), and arabinose (Ara). WSPs samples (3 mg) were added to 1 mL of 4 M trifluoroacetic acid (TFA) solution in a sealed flask filled with N_2_ at 100°C and hydrolyzed for 4 h. Excess TFA in the samples was removed by repeated evaporation with absolute ethanol at 50°C. Subsequently, 50 μL of prepared hydrolysate samples or monosaccharide standard were added to 50 μL of 1-phenyl-3-methyl-5-pyrazolone (PMP, 0.5 M) solution and 50 μL of NaOH solution (0.3 M) at 70°C for 50 min. The supernatant from centrifugation was mixed with 50 μL of HCl (0.3 M). Finally, 1 mL of chloroform was used to remove the excess PMP, and this step was repeated three times. The aqueous layer was filtered by a 0.22 μm membrane before HPLC analysis. The analytes were separated using the Agilent 1260 HPLC instrument (Agilent Technologies, Santa Clara, CA, USA) equipped with Eclipse Plus reverse C18 column (4.6 × 250 mm, 5 μm, Agilent), at a constant flow rate of 0.4 mL/min and monitored by UV-detection (245 nm). The column oven temperature was set to 35°C. A linear gradient of ammonium acetate in water (50 mM, pH 4.5, solvent A) and acetonitrile (solvent B) was applied, and the elution procedure is shown in Supplementary Table 1.

#### Fourier transform infrared spectrometer analysis

The chemical structure of three WSPs fractions from agro-food wastes was characterized by Fourier transform infrared (FTIR). FTIR was performed using IR Affinity-1S, SHIMADZU equipment (Kyoto, Japan) over a wave-number range of 400–4,000 cm^–1^. The target products were examined using the KBr pellet technique.

### Determination of *in vitro* antioxidant activity of water-soluble polysaccharides

#### Determination of 1,1-diphenyl-2-picrylhydrazyl radical scavenging activity

The scavenging ability of three WSPs fractions on DPPH radical (1,1-diphenyl-2-picrylhydrazyl free radical) was measured in accordance with the previously described method (35). In brief, 1 mL of DPPH (0.1 mM) and 1 mL of WSPs (1–5 mg/mL) were mixed at 25°C for 0.5 h in dark. Ascorbic acid (Vc) was used as a positive control. The solutions lacking WSPs or DPPH were designated as the blank group and control group, respectively. The absorbance of the solution was then measured at 517 nm. DPPH scavenging activity was calculated using Eq. (1).


(1) $$\text{Scavenging ability(\%)}=\left[1-\frac{A2-A1}{A0}\right]\times 100\%$$

where A0 is the absorbance of the blank group, and A1 and A2 are the absorbance of the control and specimen groups, respectively.

#### Determination of 2,2-azinobis-(3-ethylbenzthiazoline-6-sulphonate) radical scavenging activity

The scavenging ability of three WSPs fractions on ABTS radical (2,2-azinobis-(3-ethylbenzthiazoline-6-sulphonate) was measured as described by Meng et al. (36) with minor modifications. Briefly, the ABTS radical solution was prepared by adding 2.6 mM of K_2_S_2_O_8_ to 7 mM ABTS solution and incubated in dark for 16 h. 0.1 mL of WSPs (1–5 mg/mL) were mixed with 3.9 mL of the diluted ABTS radical solution and incubated at room temperature in dark for 10 min. The absorbance at 734 nm was measured. The ABTS scavenging activity was calculated using Eq. (1) above.

#### Determination of OH radical scavenging activity

The scavenging ability of three WSPs fractions on OH radical was measured by the Fenton method (37). Hydroxyl radical solutions were prepared by mixing 1 mL of H_2_O_2_ (9 mM), 1 mL of FeSO_4_ (9 mM), and 0.5 mL of salicylic acid–ethanol solution (9 mM). One milliliter of WSPs (1–5 mg/mL) was mixed with prepared hydroxyl radical solutions and measured at 510 nm absorbance. The scavenging ability of WSPs was calculated using Eq. (1) above.

#### Determination of ferric-reducing power

Ferric-reducing power (FRAP) of three WSPs fractions was determined according to the previous report (38) with minor modifications. Various concentrations (1–5 mg/mL) of WSPs were prepared with 0.2 mM PBS (pH 6.6). The reaction solution containing 1 mL of WSPs solution, 1 mL of 1% K_3_Fe(CN)_6_, and 1 mL of 0.2 mM PBS (pH 6.6) buffer was incubated at 50°C for 20 min. One milliliter of 10% trichloroacetic acid was added to quench the reaction. One milliliter of the supernatant was added to 1 mL of 0.1% FeCl_3_ solution and incubated at room temperature for 30 min in dark. The absorbance at 700 nm was measured. The ferric-reducing power of WSPs was calculated using Eq. (2).


(2) Reducing power=A1−A2

where A1 and A2 are the absorbance of the specimen and blank group, respectively.

#### Determination of superoxide anion scavenging activity

The scavenging ability of three WSPs fractions on superoxide anion radical (O_2_) was investigated as described by Cheng et al. (39) with a minor modification. Briefly, Tris–HCl buffer (50 mM, pH 8.2), pyrogallol (25 mM), and WSPs (1–5 mg/mL) were mixed at 25°C for 5 min. The absorbance at 299 nm was measured to determine the superoxide anion scavenging ability using Eq. (3).


(3) $$\text{Scavenging ability(\%)}=\left[\frac{A1-A2}{A1}\right]\times 100\%$$

where A1 and A2 are the absorbance of the blank and specimen group, respectively.

#### Orthogonal test design of co-culture fermentation conditions for water-soluble polysaccharides production

In order to optimize the co-culture fermentation conditions for WSPs production, an orthogonal test design was conducted. Fermentation time (3, 4, 5, 6, and 7 days), fermentation temperature (28°C, 31°C, 34°C, 37°C, and 40°C), strain proportion [*Photobacterium* sp. LYM-1 (PsL): *A. pullulans* 2012 (AP), 1:3, 1:2, 1:1, 2:1, and 3:1], and the inoculum concentration (4, 6, 8, 10, and 12%) were all tested in single-factor experiment. The orthogonal experiments were carried out with four factors and three levels on the basis of fundamental single-factor tests as shown in Supplementary Table 2. The yield (%) of WSPs was the dependent variable.

## Results and discussion

### Screening and identification of strains

**FIGURE 1:**
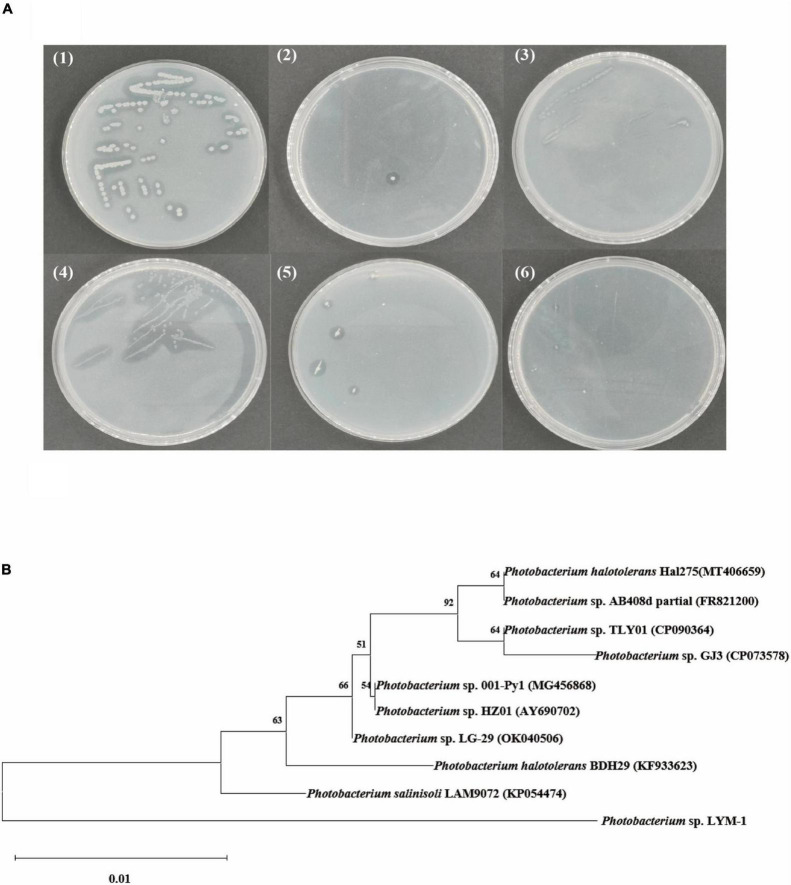
**(A)** Screening of strains: (1) *Photobacterium* sp. LYM-1, (2) *Aeromonas* sp. ZXY-3, (3) *Vibrio* sp. WM-1, (4) *Rheinheimera* sp. ZXY-2, (5) *Shewanella* sp. ZXY-1, and (6) *Pseudomonas* sp. LYM-2; **(B)** neighbor-joining tree based on 16S rRNA gene sequences showing the phylogenetic relationships between strain *Photobacterium* sp. LYM-1 and its related taxa.

Strain ability to degrade chitin was determined by examining the size of the transparent circle on the colloidal chitin screening plate as shown in Figure 1. The one showing the largest transparent circle was selected as the experimental strain. The type of the strain was identified by 16S rRNA gene sequencing and further comparison in the NCBI database. In this study, the strain identified as *Photobacterium* sp. (named *Photobacterium* sp. LYM-1) was the best microorganism for utilizing shrimp shells (Figure 1A1). The phylogenetic tree in Figure 1B shows that the bacterium *Photobacterium* sp. LYM-1 is most closely related to *Photobacterium* sp. LAM9072. *Photobacterium* sp. LAM9072, a Gram-stain-negative, aerobic, motile, rod-shaped bacterium, was first identified to degrade sulfonylurea herbicides, a type of drug residue in soil (40).

### Compatibility of *Photobacterium* sp. LYM-1 and *Aureobasidium pullulans* 2012

**FIGURE 2:**
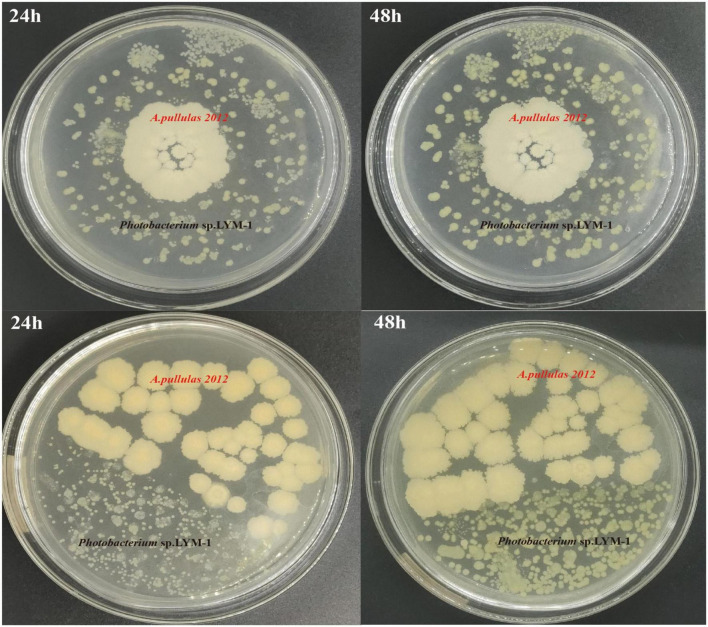
Compatibility result of *Photobacterium* sp. LYM-1 and *A. pullulans* 2012.

An antagonistic test between *Photobacterium* sp. LYM-1 and *A. pullulans* 2012 revealed that there was no antagonism between them (Figure 2). After 24 h, the two strains grew at a similar rate and no bacteriostatic zone was observed in either strain. After 48 h, these two strains could fuse with no discernible boundaries. This result demonstrated that these two strains could coexist and grow harmoniously. Co-culture fermentation of yeast and bacteria has been successfully applied to other products (41).

### Chemical components of water-soluble polysaccharides

Both straw and shrimp shells are examples of agro-food wastes. Straw is rich in carbohydrates and shrimp shells contain carbohydrates, protein, and other nutrients. However, the carbohydrates (cellulose in straw and chitin in shrimp shells) are not water soluble, which limits their application. The WSPs production by the fermentation of these two materials could be viable, green, and high-value utilization strategy (22, 42). Monocultures and co-culture fermentations of *Photobacterium* sp. LYM-1 and *A. pullulans* 2012 for the production of WSPs were investigated in this study. Three types of WSPs (PsL-WSPs, AP-WSPs, and PsL/AP-WSPs) were obtained by fermentation broth collection, ethanol precipitation, deproteinization, and dialysis.

Table 1 summarizes the content of saccharides, uronic acid, protein, flavone, polyphenol, and ash of WSPs. Saccharides make up the majority of all three fractions, particularly the WSPs fraction from co-culture fermentation (64.88% of saccharides). *A. pullulans* is the type strain for the production of the polysaccharide, especially pullulan (20). Co-culture of *Photobacterium* sp. LYM-1 and *A. pullulans* 2012 could produce more WSPs, indicating that these two strains have a mutual promotion on the WSPs production, which is consistent with the compatibility test result. Cellulolytic enzyme production by co-cultured of *Trichoderma reesei* and *Aspergillus niger* in solid substrate fermentation using alkali-treated sugar cane bagasse as substrate was reported (43), demonstrating that co-culture of agro-food waste could be used for the preparation of high-value products. Co-culture fermentation of yeast and bacteria has also been used successfully in other applications (41). Uronic acid was found in all three WSPs fractions, with the highest concentration found in PsL/AP-WSPs, reaching 12.97%. Although proteins were detected in all three WSPs fractions, their content (1.31% in PsL/AP-WSPs, 0.82% in PsL-WSPs, and 0.50% in AP-WSPs) could be ignored in this study when compared to saccharides contents. AP-WSPs contained more flavonoids (14.81%) compared to the other two WSPs fractions. The polyphenol and ash contents in the three WSP fractions were very low and did not differ significantly.

**TABLE 1:** Chemical components of PsL/AP-WSPs, PsL-WSPs, and AP-WSPs.

Samples	PsL/AP-WSPs	PsL-WSPs	AP-WSPs
Saccharides yield (%)	64.88 ± 3.21^a^	53.58 ± 2.13^c^	58.28 ± 2.45^b^
Uronic acid content (%)	12.97 ± 1.13^a^	5.80 ± 0.21^c^	10.47 ± 1.09^b^
Protein content (%)	1.31 ± 0.26^a^	0.82 ± 0.20^b^	0.50 ± 0.23^b^
Flavone content (%)	8.01 ± 0.43^b^	7.03 ± 0.14^b^	14.81 ± 2.22^a^
Polyphenol content (%)	2.20 ± 0.15^b^	2.43 ± 0.24^b^	3.05 ± 0.11^a^
Ash content (%)	3.21 ± 0.32^b^	3.83 ± 0.56^a^	3.47 ± 0.42^ab^

Values with the different superscript letters in the same line indicate that they are statistically different (*p* < 0.05). The values are presented as mean ± SD (*n* = 3).

### Molecular weight of water-soluble polysaccharides

**FIGURE 3:**
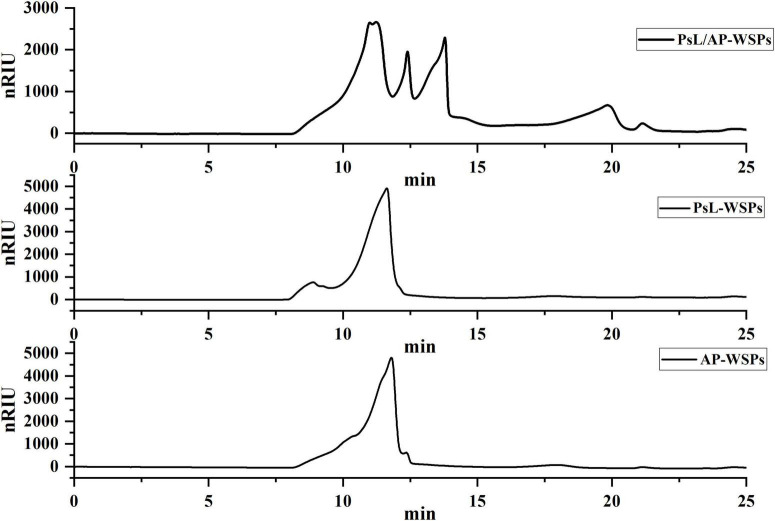
HPLC profiles of PsL/AP-WSPs, PsL-WSPs, and AP-WSPs.

In general, the molecular weight of polysaccharides produced through fermentation is highly centralized. The molecular weight distributions of three WSPs fractions are shown in Figure 3. Only a distinct group peak was observed in PsL-WSPs and AP-WSPs fractions with a similar molecular weight of 257.6 and 227.6 kDa, respectively, quantified by molecular weight regression equation (log M_*W*_ = 0.326t+9.202, *R*^2^ = 0.9947) showing in Supplementary Figure 1 (9). The characteristic peaks of PsL/AP-WSPs were adjacent and complex, with distinct differences from those of PsL-WSPs and AP-WSPs. The molecular weights of PsL/AP-WSPs were 414.9, 346.8, 144.6, and 50.9 kDa, which could be attributed to the synergistic effect of complex enzymes (e.g., cellulose, chitinase, and deacetylase) expressed by *Photobacterium* sp. LYM-1 and *A. pullulans* 2012. Similar phenomena were also reported in previous studies (26, 44).

### Monosaccharide composition of water-soluble polysaccharides

The monosaccharide composition of three WSPs fractions was analyzed by HPLC. Monosaccharide analysis showed that they were heteropolysaccharides composed primarily of Man, Rib, GlcN, Glc, Gal, and Ara in different molar percentages (as shown in Supplementary Figure 2 and Table 2). Results revealed that PsL/AP-WSPs and PsL-WSPs comprised the same monosaccharides in different molar ratios. Gal (16.38%) was the major monosaccharide structure of PsL/AP-WSPs, while Glc (19.20%) and GlcN (14.52%) were the most common monosaccharides of PsL-WSPs, possibly due to chitin degradation by *Photobacterium* sp. LYM-1 to its monomer *N*-acetylglucosamine as the main carbon source instead of Glc. However, Rib was not detected in AP-WSPs, and the proportion of Rib in PsL/AP-WSPs was higher, owing to the benefit of co-culture fermentation (44).

**TABLE 2:** Monosaccharide composition of PsL/AP-WSPs, PsL-WSPs, and AP-WSPs.

Monosaccharide	PsL/AP-WSPs		PsL-WSPs		AP-WSPs	
			
	Mole ratio	%	Mole ratio	%	Mole ratio	%
Mannose	0.443	8.10%	0.436	7.98%	0.451	8.25%
Ribose	0.388	8.28%	0.129	2.75%	–	–
Glucosamine	0.140	3.41%	0.594	14.52%	0.252	6.15%
Glucose	0.503	13.39%	0.722	19.20%	0.734	19.52%
Galactose	0.571	16.38%	0.365	10.49%	0.452	12.98%
Arabinose	0.445	13.61%	0.325	9.94%	0.339	10.37%

– Means not detected.

### Fourier transform infrared spectrometer analysis

**FIGURE 4:**
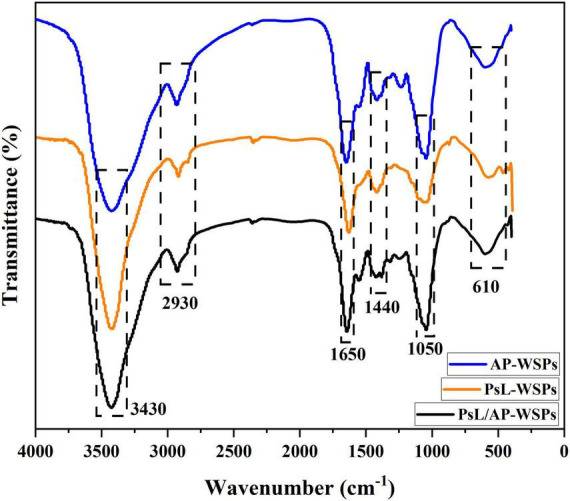
FTIR characterization of AP-WSPs, PsL-WSPs, and PsL/AP-WSPs.

Fourier transform infrared (FTIR) spectrometer result of AP-WSPs, PsL-WSPs, and PsL/AP-WSPs is shown in Figure 4. Spectra peaks at 3,430, 2,930, 1,650, 1,440, 1,050, and 610 cm^–1^ were the typical characteristic of polysaccharides (45). There is a strong and wide stretching peak around 3,430 cm^–1^, which is caused by the stretching vibration of hydroxyl groups, the basic FTIR peak of polysaccharides (35). The weak absorption peak around 2,930 cm^–1^ was assigned to the C-H stretching vibration of -CH_3_, -CH_2_, and -CH of polysaccharides. The band at close to 1,650 cm^–1^ was attributed to the asymmetric stretching vibration of the carbonyl group (46). C-H angular vibration caused the characteristic peak around 1,440 cm^–1^. The strong absorption peak at 1,000–1,200 cm^–1^ is dominated by stretching vibrations of ring structures, C-C, and deformation of -CH_2_ groups vibration characteristic of polysaccharide (47). The discussed characteristic FTIR spectra peaks demonstrated that AP-WSPs, PsL-WSPs, and PsL/AP-WSPs obtained from the monoculture and co-culture fermentation of straw and shrimp shells were polysaccharides.

### Antioxidant activities of water-soluble polysaccharides

#### 1,1-diphenyl-2-picrylhydrazyl radical scavenging ability

**FIGURE 5:**
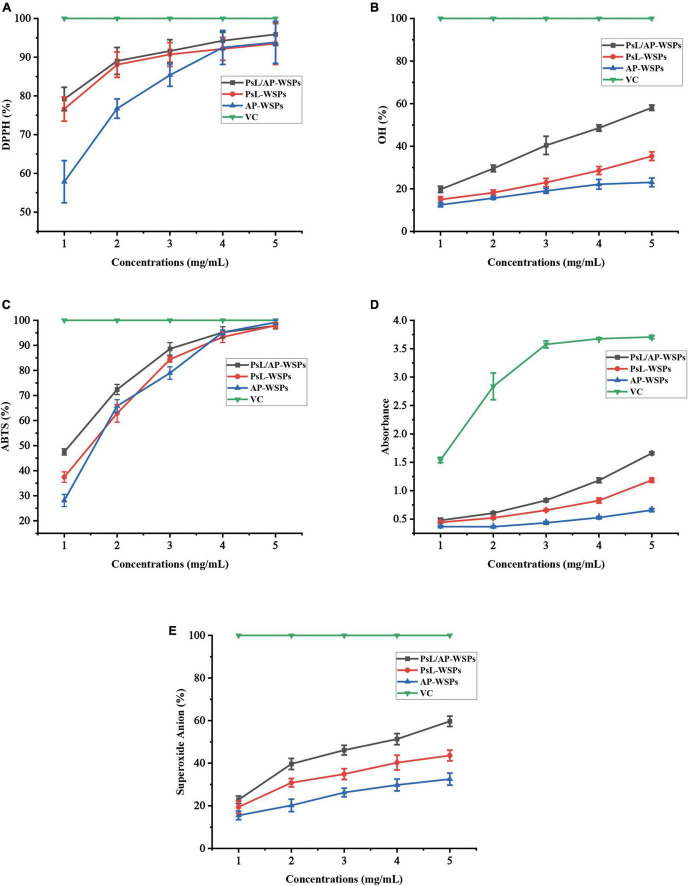
Evaluation of antioxidant activities of WSPs. **(A)** DPPH radical scavenging ability, **(B)** OH radical scavenging ability, **(C)** ABTS radical scavenging ability, **(D)** ferric-reducing antioxidant power, and **(E)** superoxide anion scavenging ability.

Excess free radicals in the human body can cause various adverse reactions, such as aging, obesity, and diabetes, whereas, polysaccharides can improve immune function, antioxidation, and exhibit low cellular toxicity (48). DPPH radical has been widely used as an indicator to estimate the free radical scavenging activities of antioxidants because of its high sensitivity, fast reaction, and stability (11). The three WSP fractions demonstrated remarkable DPPH radical scavenging activities, as shown in Figure 5A, which is consistent with previous research (49). PsL/AP-WSPs (∼94%) showed higher DPPH radicals scavenging activity than the other two fractions (∼90%) and the polysaccharides GPs1 (∼68%) from *Glycyrrhiza uralensis* at 4 mg/mL (50). The monosaccharide composition of the three WSPs fractions might cause differences in the glucosidic bonds, configurations, and sequence of monosaccharides, and have a major influence on their hydrogen-donating abilities (51). The PsL/AP-WSPs superior scavenging ability against the DPPH radical compared to the other two WSPs fractions might be attributed to its strong hydrogen-donating ability and uronic acid groups (52, 53).

#### Hydroxyl radical scavenging ability

The ability of hydroxyl radical scavenging abilities of three WSPs fractions is shown in Figure 5B. Three WSPs fractions demonstrated a concentration-dependent scavenging ability against hydroxyl radicals within the determined dosage range. Furthermore, in each determination concentration, PsL/AP-WSPs demonstrated a greater ability to scavenge hydroxyl radicals than the other two WSP fractions. At the concentration of 2 mg/mL, the hydroxyl radical scavenging capacity of PsL/AP-WSPs (∼25%) was higher than that of GPN (24%) from *G. glabra* (54).

#### 2,2-azinobis-(3-ethylbenzthiazoline-6-sulphonate) radical scavenging ability

2,2-azinobis-(3-ethylbenzthiazoline-6-sulphonate) scavenging experiment has been widely used to assess the antioxidant capacity of various natural products, including, polyphenols, flavonoids, and polysaccharides (55). As shown in Figure 5C, the ABTS radical scavenging activity of three WSPs fractions increased in a quadratic concentration-dependent pattern. At low concentrations (1–4 mg/mL), the ABTS radical scavenging abilities differed in the following order: PsL/AP-WSPs > PsL-WSPs > AP-WSPs. However, in each determination concentration, all three WSPs fractions exhibited higher ABTS radical ability than that of FWPS1-1 from *Fritillaria unibracteata var. wabuensis* (56). At a concentration of 5 mg/mL, the ABTS radical scavenging activities of the three WSPs showed a similar scavenging level of V_*C*_, indicating that WSPs possessed notable ABTS radical scavenging ability.

#### Ferric-reducing antioxidant power

Ferric-deducing power (FRAP) assay is widely used to estimate the antioxidant capacity of polysaccharides (57). In this study, all three WSPs showed apparent FRAP within the elevated concentration of WSPs, indicating that WSPs were electron donors and could quench the radical chain reactions (Figure 5D). In the determination concentration, the FRAP of three WSPs followed the order of PsL/AP-WSPs > PsL-WSPs > AP-WSPs.

#### Superoxide anion scavenging ability

Superoxide anion radicals have a lower reactivity and a longer lifetime than other free radicals (58). As a result, the scavenging effect of superoxide anion radicals is an essential experimental index for the determination of antioxidant activity (59). As shown in Figure 5E, all three WSPs fractions have superoxide anion radical scavenging activity, with increasing capacity proportional to WSPs concentrations. The capacity of the three WSPs fractions was as follows: PsL/AP-WSPs > PsL-WSPs > AP-WSPs, which showed a free radical scavenging trend similar to the other free radical reagents.

### Optimization of water-soluble polysaccharides production by orthogonal design

#### Effect of fermentation time on polysaccharides yield

**FIGURE 6:**
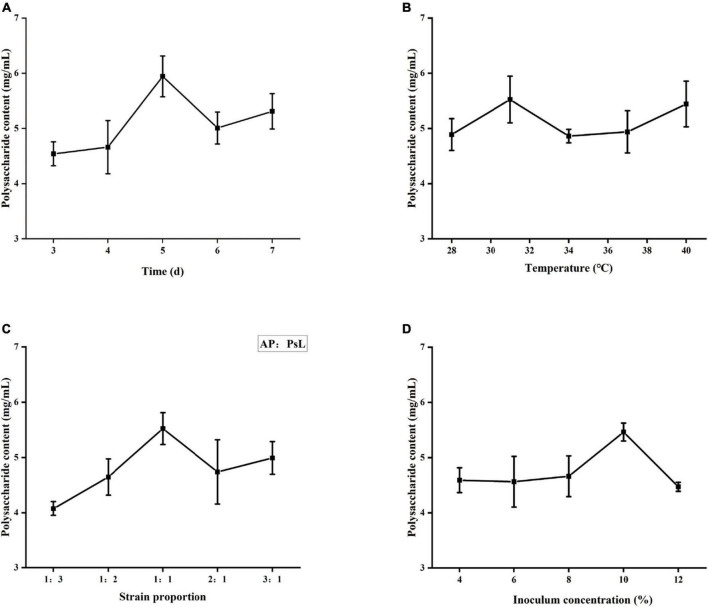
Effects of different factors on polysaccharide yield. **(A)** Fermentation time, **(B)** fermentation temperature, **(C)** strain proportion, and **(D)** inoculum concentration.

When the chemical components, molecular weights, monosaccharide composition, and antioxidant capacity of three WSP fractions were combined, PsL/AP-WSPs demonstrated the best characteristics. Co-cultured microorganisms could be beneficial because of the synergistic expression of the microorganisms’ metabolic pathways. The strategy for improving the production of PsL/AP-WSPs was conducted by the orthogonal experimental design. The single-factor analysis results are shown in Figure 6. The concentration of WSPs increased slowly and then fast from 1 to 5 days of the fermentation period and the highest concentration of WSPs was observed at 5 days (Figure 6A). Fewer nutrients (e.g., yeast extract and glucose) in the fermentation medium resulted in low WSPs production by these two organisms at an early period. As straw and shrimp shells were hydrolyzed by secreted enzymes (such as cellulases, chitinases, and proteases) for nutrients, they grew rapidly and produced extra polysaccharides, leading to the high polysaccharides concentration in the fermentation broth (20). According to the effect of fermentation time on the yield of WSPs, the factor levels of feeding time were chosen for 4, 5, and 6 days.

#### Effect of fermentation temperature on polysaccharide yield

Each organism requires a specific optimum temperature for reproduction. Extreme temperatures will affect enzyme activities and metabolism and eventually lead to death. As shown in Figure 6B, the concentration of WSPs in fermentation broth increased with temperature from 28 to 31°C and then decreased when the temperature was increased to 31°C. The optimum fermentation temperature for the production of PsL/AP-WSPs was 31°C. Our findings are consistent with the previous studies relating to the growth temperatures of *Aureobasidium* sp. and *Photobacterium* sp. (60, 61). According to the effect of temperature on the yield of WSPs, the factor levels for choosing the fermentation temperature were 28, 31, and 34°C.

#### Effect of strain proportion on polysaccharide yield

On account of several microbial interactions, the growth and the metabolite of monoculture differ from that of mixed culture (62). As shown in Figure 6C, the highest PsL/AP-WSPs concentration in the fermentation broth was observed when the equivalent strain proportion (1:1) of *A. pullulans* 2012 (AP) with *Photobacterium* sp. LYM-1 (PsL) was used. It can be explained as the phenomenon of mutual competition and interdependence among different strains (41). The microbial interactions can be antagonistic or synergistic, resulting in inhibited or enhanced proliferation, respectively. In this study, *A. pullulans* 2012 showed that synergistic interaction to *Photobacterium* sp. LYM-1 results in more extracellular polysaccharides in their equivalent proportion. Therefore, the factor levels for choosing the strain proportion were 1:2, 1:1, and 2:1.

#### Effect of inoculation amount on polysaccharide yield

As shown in Figure 6D, the PsL/AP-WSPs concentration increased in the elevated inoculation concentration of 4–10%, and then slightly decreased over 10% of the inoculation concentration. The hypothesis is that the increased concentration of cultures consumed more nutrients and resulted in insufficient dissolved oxygen, inhibiting culture growth and metabolism and resulting in less metabolites, including WSPs (63). According to the influence of inoculation amount on WSPs yield, the factor levels for choosing the inoculation amount were 8, 10, and 12%.

#### Orthogonal experimental results

The influences of the fermentation factors on the indicator were determined using range (R) analysis. According to the orthogonal experiment in Table 3, the influence of each factor on the polysaccharide content was in the following order: **B** > **D** > **C** > **A**. The fermentation temperature had the greatest impact on the polysaccharide yield, followed by inoculation amount, strain ratio, and time. According to the analysis, the combination **A**_1_**B**_2_**C**_3_**D**_2_ was optimal, which indicated that the optimum conditions for the production of PsL/AP-WSPs were 5 days, at 31°C, inoculation concentration of 10%, and 2:1 (AP: PsL) strains ratio. As the combination of **A**_1_**B**_2_**C**_3_**D**_2_ did not appear in the orthogonal test design, a verification test with three parallel experiments was carried out. The yield of polysaccharides was 5.88 ± 0.40 mg/mL, which was higher than other conditions. Therefore, it was confirmed that **A**_1_**B**_2_**C**_3_**D**_2_ provided the best fermentation conditions.

**TABLE 3:** The L_9_ (3^4^) orthogonal experiment design and results.

Test number	Factor				
	
	A Time (d)	B Temperature (°C)	C Strain proportion	D Inoculum concentration (%)	Polysaccharide concentration (mg/mL)
1	1	1	1	1	4.65
2	1	2	2	2	5.59
3	1	3	3	3	5.25
4	2	1	2	3	4.40
5	2	2	3	1	5.56
6	2	3	1	2	5.14
7	3	1	3	2	4.87
8	3	2	1	3	5.31
9	3	3	2	1	5.08
*K*_1_	5.167	4.642	5.035	5.099	
*K*_2_	5.036	5.489	5.026	5.203	
*K*_3_	5.088	5.160	5.230	4.989	
*R*	0.131	0.846	0.204	0.213	

The arrangements of columns **A**, **B**, **C,** and **D** were decided by orthogonal design for 3 (factor) * 9 (run number); every row of the run number represents one experimental replicate; every run was carried out triplicate (*n* = 3, mean ± SD).

EPSs and their production from agro-industrial waste have been extensively researched (20, 64). Sugarcane bagasse hydrolysate and jackfruit seed powder were used for the production of pullulan by *A. pullulans* in shake-flask fermentations at 28°C for 4 days, with a yield of 25.19 and 18.76 g/L, respectively, which was higher than that from straw and shrimp shell (5.88 g/L) (65, 66). However, in the reported studies, almost all the fermentation media contained nutrients, such as yeast extract and peptone, except for agro-food wastes. Whereas, the fermentation media for mono- and co-cultures of *Photobacterium* sp. LYM-1 and *A. pullulans* 2012 only contained straw and shrimp shells for nutrients, which reduced the costs.

## Conclusion

In this study, the strain *Photobacterium* sp. LYM-1, isolated from the mudflat containing shrimp shell, was identified to degrade chitin for the first time. *Photobacterium* sp. LYM-1 and *A. pullulans* 2012 are compatible and could be co-cultured using shrimp shells and straw as a substrate for the production of WSPs. Co-culture and monoculture fermentations of *Photobacterium* sp. LYM-1 and *A. pullulans* 2012 yielded three types of WSP fractions (PsL/AP-WSPs, PsL-WSPs, and AP-WSPs). All three WSPs fractions were heteropolysaccharides, primarily composed of mannose, ribose, glucose, glucosamine, galactose, and arabinose in varying molar ratios and exhibited significant antioxidant activity. However, PsL/AP-WSPs outperformed the other two WSPs fractions in terms of characteristics and antioxidant activity. The high antioxidant activity of PsL/AP-WSPs could be attributed to their compositional diversity and higher uronic acid content. The production conditions of PsL/AP-WSPs were optimized by orthogonal design and the optimum fermentation conditions were determined to be 5 days fermentation time, at 31°C, in a 2:1 AP to PsL ratio, and inoculation amount of 10%. The highest yield of PsL/AP-WSPs was 5.88 mg/mL under optimum fermentation conditions. In conclusion, this research demonstrated that preparing PsL/AP-WSPs from agro-food wastes using the co-culture fermentation of *Photobacterium* sp. LYM-1 and *A. pullulans* 2012 were feasible, economical, and environmentally friendly.

## Data availability statement

The data presented in the study are deposited in: https://figshare.com/articles/dataset/Raw_Data_of_the_manuscript_1047932_zip/21563475. The 16S rRNA information is deposited in: https://www.ncbi.nlm.nih.gov/. GenBank accession numbers are: OP824621; OP824622; OP824623; OP824624; OP824625; and OP824626. Further inquiries can be directed to the corresponding author.

## Author contributions

YL: conceptualization, resources, supervision, project administration, writing—review and editing, and funding acquisition. MWa: data curation, writing—original draft, and funding acquisition. YZ and XZ: data curation and software. XL: software and methodology. FL and DW: methodology and writing—original draft. MWe: methodology. XY: investigation, data curation, project administration, writing—review and editing, and funding acquisition. All authors contributed to the article and approved the submitted version.

## Footnotes

1
www.ncbi.nlm.nih.gov/blast

## Abbreviations

WSPs: water-soluble polysaccharides
PsL/AP-WSPs: WSPs from co-culture fermentation of *Photobacterium* sp. LYM-1 and *A. pullulans* 2012
PsL-WSPs: WSPs from monoculture fermentation of *Photobacterium* sp. LYM-1
AP-WSPs: WSPs from monoculture fermentation of *A. pullulans* 2012
FTIR: Fourier transform infrared spectra
EPS: microbial exopolysaccharides
RID: refractive index detector
NCBI: National Center for Biotechnology Information
Gal: galactose
Man: mannose
Fuc: fucose
GlcN: glucosamine
Glc: glucose
GalA: galacturonic acid
Rha: rhamnose
Ara: arabinose
FRAP: ferric-reducing antioxidant power
TFA: trifluoroacetic acid
PMP: 1-phenyl-3-methyl-5-pyrazolone
DPPH: 1,1-diphenyl-2-picrylhydrazyl
ABTS: 2,2-azinobis-(3-ethylbenzthiazoline-6-sulphonate)
SmF: submerged fermentation
SSF: solid-state fermentation
HW: hot-water extraction
UA: ultrasonic-assisted extraction
ME: microwave-assisted extraction
EA: enzyme-assisted extraction
EUA: enzyme-ultrasonic assistance extraction
HPLC: high-performance liquid chromatography.
