# Supplementary material for: Antioxidant properties of water-soluble polysaccharides prepared by co-culture fermentation of straw and shrimp shell
Source: Front Nutr. 2022 Nov 21;9:1047932. doi: 10.3389/fnut.2022.1047932 (PMC9720685; doi:10.3389/fnut.2022.1047932)
Supplement: Supplementary file 1 [file Data_Sheet_1.PDF]

Supplementary Data

Antioxidant Properties of Water-Soluble Polysaccharides Prepared by Co-Culture Fermentation of Straw and Shrimp Shell

Yongmei Lyu[1,2 †], Mian Wang[1†], Yiwen Zhang[1], Xiaoyang Zhang[1], Xiaochen Liu[1], Fengwei Li[1], Dujun Wang[1], Ming Wei[1], Xiaohong Yu [1*]

1 School of Marine and Bioengineering, Yancheng Institute of Technology, Yancheng 224051, China;

2 Jiangsu Key Laboratory of Marine Bioresources and Environment, Jiangsu Ocean University, China.

***Correspondence:**
yxh1127@ycit.edu.cn (X. Yu)

†These authors contributed equally.

Supplementary Table 1 The procedure of gradient elution of LC.

Time (min)	flow rate (mL/min)	solvent A (%)	solvent B (%)
0	0.4	81	19
28	0.4	73	27
50	0.4	5	95
51	0.4	5	95
52	0.4	81	19
60	0.4	81	19

Supplementary Table 2 Orthogonal experimental design L_9 (3^4).

level	Factor			
	**A**<br>Fermentation time (d)	**B**<br>Fermentation temperature (°C)	**C**<br>Strain proportion	**D**<br>Inoculum concentration (%)
1	5	28	1:2	8
2	6	31	1:1	10
3	7	34	2:1	12

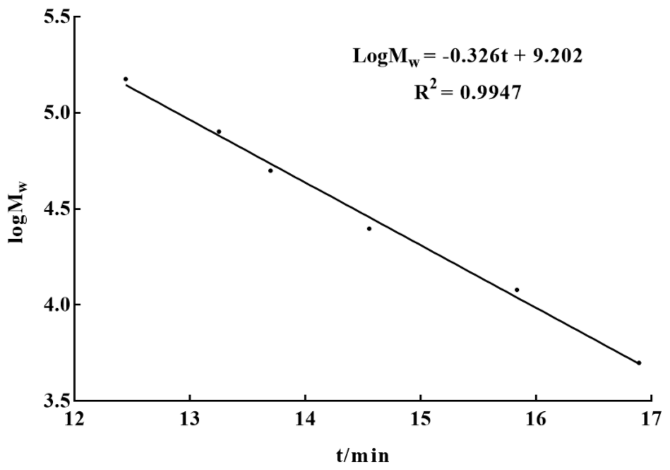

Supplementary Figure 1 Standard curve of dextran molecular weight.

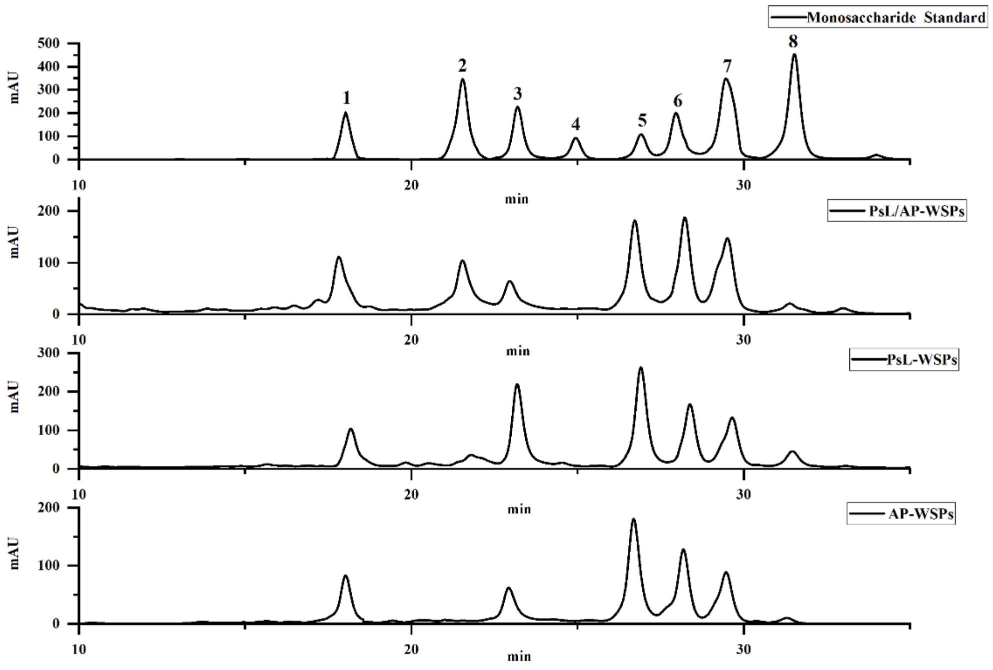

Supplementary Figure 2 Monosaccharide composition of PsL/AP-WSPs, PsL-WSPs, AP-WSPs (1. Mannose, 2. Ribose, 3. Glucosamine, 4. galacturonic acid, 5. Glucose, 6. Galactose, 7. Arabinose, 8. Fucose).
